# Impact of Mediterranean diet–based nutritional intervention on gluten-free diet, nutritional status, and quality of life in a pediatric celiac population: a randomized controlled trial

**DOI:** 10.3389/fnut.2026.1878608

**Published:** 2026-08-28

**Authors:** Farah Faiza Kaddour, Nadia Mahdad, Karim Bouziane Nedjadi, Malika Bouchenak

**Affiliations:** 1 Laboratory of Clinical and Metabolic Nutrition, Faculty of Nature and Life Sciences, University of Oran 1 Ahmed Ben Bella, Oran, Algeria; 2 Department of Pediatrics, University Hospital Center of Oran, Faculty of Medicine, University of Oran 1 Ahmed Ben Bella, Oran, Algeria

**Keywords:** gluten-free diet, Mediterranean diet, nutritional intervention, pediatric celiac population, quality of life

## Abstract

**Introduction:**

Although strict adherence to gluten-free diet (GFD) is essential for celiac disease (CeD) management, it does not necessarily ensure optimal or nutritionally adequate diet and lifestyle.

**Objective:**

To assess the impact of combining Mediterranean diet (MD)–based nutritional education, and psychological and culinary workshops on adherence to GFD, dietary quality, physical activity (PA), and quality of life (QoL) in children and adolescents with CeD.

**Methods:**

A randomized controlled trial was conducted in 59 celiac children and adolescents (aged 5–18 years), allocated into two groups. The intervention group (IG, *n* = 35) received standard therapeutic education plus MD–based nutritional education, psychological support sessions, and cooking workshops. The control group (CG, *n* = 24) received only standard therapeutic education. Anthropometric measurements, biochemical parameters, MD (KIDMED index) and GFD (CDAT) adherence, PA, CeD-specific QoL (CDDUX), health-related (HR) QoL (KIDSCREEN-10), and nutritional intakes were assessed at baseline and at day 90. A linear mixed- model was used to assess the effect of group, time, and their interaction on MD adherence. A multivariate linear regression analysis was performed to identify independent predictors of change in MD adherence (ΔMD).

**Results:**

At day 90, IG exhibited higher MD adherence (*p* < 0.001), better GFD adherence (*p* = 0.002), and improved lipid quality (*p* < 0.05), compared with CG. Moreover, increased intakes of fiber (*p* = 0.010) and calcium (*p* < 0.001) were noted. Reduced sedentary time (*p* = 0.005), and higher CDDUX “GFD domain” scores (*p* = 0.05) were observed. At day 90 in IG, significant improvements were observed for KIDMED index GFD adherence, as well as fiber, protein and calcium intakes (*p* < 0.001). Additionally, CDDUX (*p* < 0.001), and KIDSCREEN-10 (*p* < 0.001) scores were improved compared with baseline. The linear mixed model revealed a significant group × time interaction (β = −4.42, *p* < 0.001), indicating that the change in MD adherence over time differed significantly between groups. In multivariate analysis, belonging to the IG (β = 4.52, *p* < 0.001) and having difficulty in gluten-free bread preparation (β = 1.65, *p* = 0.008) were independently associated with improvement in ΔMD.

**Conclusion:**

Integrating MD–based nutritional education into reinforced therapeutic education represents a promising strategy for the long-term management of CeD in children and adolescents.

## Introduction

Currently, a strict gluten-free diet (GFD) is the only treatment for Celiac disease (CeD), although following a GFD does not always ensure adequate nutritional status (1, 2). The management of pediatric patients with CeD is not limited to gluten restriction; studies underline the need for a broader and more practical approach to CeD management, involving dietary guidance, psychological support, and long-term follow-up (3–9). Indeed, deficiencies in key micronutrients such as iron, calcium, magnesium, and vitamins D, E, and B are frequently reported during long-term adherence to a GFD (6, 10–12). These imbalances are often linked to the frequent consumption of ultra-processed gluten-free products, which are typically rich in saturated fats and simple sugars but poor in fiber and essential micronutrients. As a result, children and adolescents following a GFD often consume diets high in fat and sugar and low in nutrient density. This occurs despite good adherence to the GFD (9, 10, 13, 14). In contrast, this dietary pattern appears less pronounced in adults, whose cereal-based food consumption and overall macronutrient intake are generally comparable to those of the general population. Nevertheless, deficiencies in vitamin D, calcium, and iron remain common despite long-term adherence to the gluten-free diet (15, 16).

Adherence is also challenged by practical and socioeconomic constraints. Gluten-free products are substantially more expensive than standard alternatives, which can limit access to balanced diets and increase the risk of food insecurity (17–19). In addition, the GFD is associated with a notable psychosocial burden, including social restrictions, limited food choices, and poor palatability (5, 20). These challenges are particularly marked during adolescence, a period in which adherence tends to decline under social pressure and in the absence of immediate symptoms. Together, these factors negatively affect quality of life (QoL) (21–24).

In this context, the Mediterranean diet (MD) can be integrated within a GFD by emphasizing naturally gluten-free foods, such as legumes, fruits, vegetables, pseudo-cereals, and sources of unsaturated fats. The MD is rich in fiber, antioxidants, and essential micronutrients, and has recognized anti-inflammatory properties (24–28). In patients with CD, such characteristics may help improve overall dietary quality, as well as, overall well-being. Indeed, higher adherence to the MD has also been associated with better QoL in celiac patients (14, 20, 24).

Overall, dietitian-led education combined with accessible food guides is crucial for management of children with CeD following a GFD (1, 29, 30). However, very few interventional studies have evaluated the impact of MD-based management in the pediatric celiac population (1, 31). Therefore, the present study aimed to evaluate the effect of a multidimensional intervention combining MD–based nutritional education, culinary workshops, and psychological support on GFD and MD adherence, dietary quality, physical activity (PA), and QoL in celiac pediatric population. To our knowledge, no randomized controlled trials have tested this approach yet in celiac patients.

## Materials and methods

### Study population

This randomized interventional study was conducted between January 2022 and January 2024 at the A. Cabral Pediatric Department “C” (CHU Oran). Eligibility criteria were defined as follows: inclusion criteria comprised children aged 5–18 years, with CeD confirmed according to ESPGHAN criteria (32), and adherence to a GFD for at least 1 year prior to enrolment. Exclusion criteria included the presence of associated nutritional disorders or neurological diseases.

A total of 145 celiac pediatric patients were recruited during routine clinical visits, and initially assessed for eligibility. Before randomization, 54 children and adolescents refused to participate due to lack of time or did not provide a reason. A total of 91 children and adolescents with CeD were randomly assigned to the intervention group (IG) (*n* = 46) and to the control group (CG) (*n* = 45), using a simple randomization. This procedure was conducted by an independent allocator (the head pediatrician of the department). Because of the nature of the intervention, this was an open-label trial, and neither participants nor the study dietitian were blinded after group allocation. To ensure allocation concealment, the study dietitian was informed of each patient’s assignment only after randomization had been completed by the pediatrician, thereby minimizing the risk of selection bias.

**FIGURE 1:**
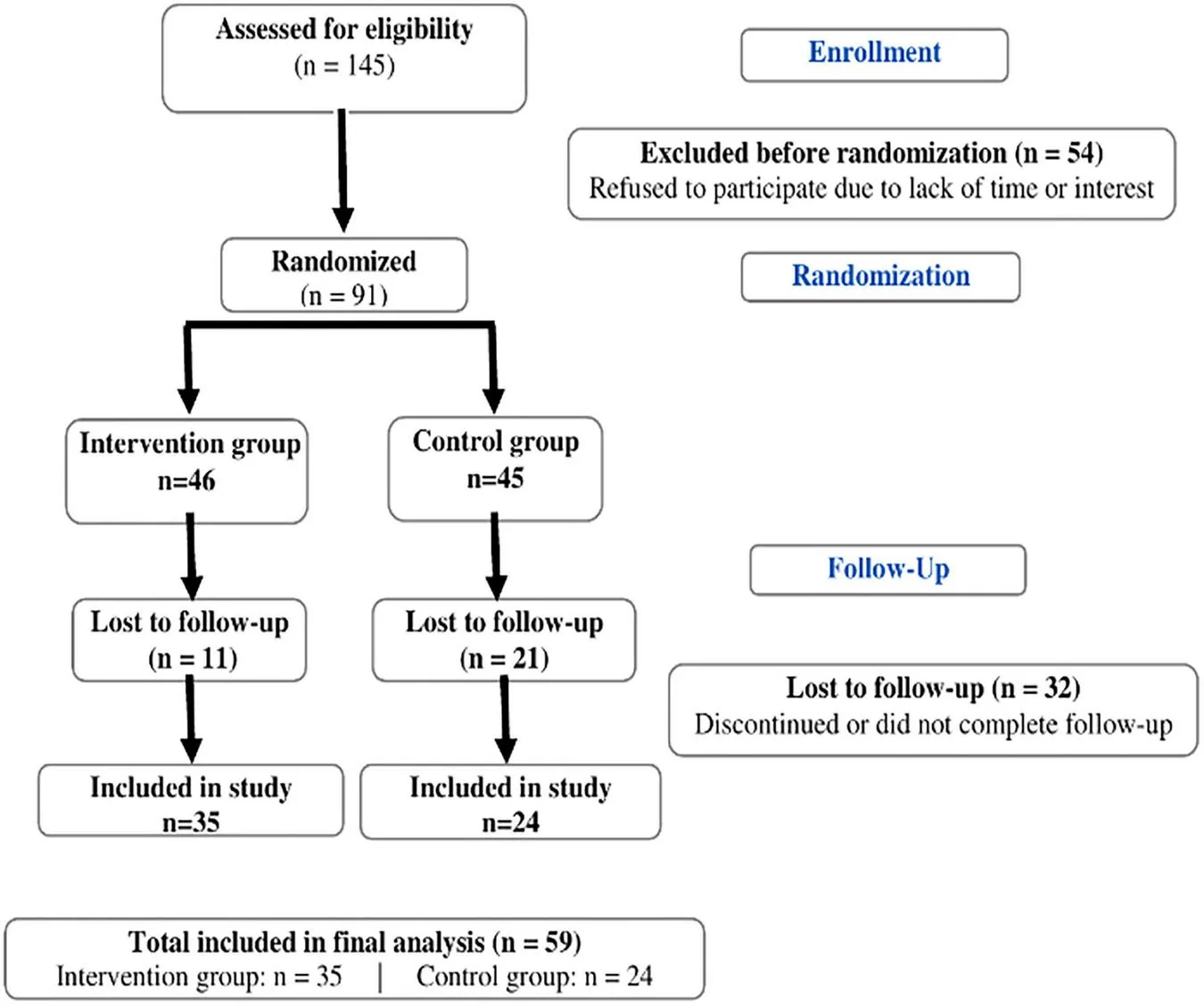
Flow diagram of participant requirement, allocation, follow-up, and analysis.

During the follow-up period, 11 patients in the IG and 21 in the CG were lost to follow-up which was mainly attributable to their unavailability during the school period, unsuccessful contact attempts, or progressive withdrawal from the study. Consequently, 59 celiac children and adolescents completed the study and were included in the final analysis: 35 in IG, who received both standardized therapeutic education and the multidisciplinary intervention, and 24 in the CG, who received only standardized therapeutic education (Figure 1).

Written informed consent was obtained from parents or legal tutor, and verbal consent was obtained from both parents and patients. The study protocol was approved by the Ethics and Deontology Committee of the Ahmed Ben Bella Oran 1 University, in accordance with ministerial decree N° 991 of December 10*^th^*, 2020.

### Baseline assessment

At the first visit (baseline, Day 0), clinical characteristics, socioeconomic status and anthropometrics parameters were determined. In addition, serum parameters, including albumin, iron status, vitamin D, calcium, mean corpuscular volume (MCV), total cholesterol and triglycerides, were analyzed. Additional details regarding biochemical and anthropometric measurements have been described previously (21).

Gluten-free diet adherence was assessed using the Celiac Dietary Adherence Test (CDAT) developed by Leffler et al. (33) and adapted for pediatric use by Pedoto et al. (34). Lower total scores indicated better adherence to GFD (≤2 = excellent adherence, 3–6 = moderate adherence, and ≥7 = poor adherence). To better characterize dietary behaviors in our population, additional study-specific questions explored voluntary gluten exposure, perceived barriers to maintaining the gluten-free diet, meal patterns, meal conviviality, and snacking habits.

Mediterranean diet adherence was evaluated using the KIDMED index (35, 36). Total scores range from −4 to 12, with higher scores indicating better adherence (≤3 = poor adherence, 4–7 = moderate adherence, and ≥8 = optimal adherence). As no validated French or Arabic versions of the CDAT and KIDMED questionnaires were available, both instruments were translated into French and Arabic by the study dietitian for use in the present study.

Dietary intake was assessed using one 24-h dietary recall combined with a 3-day food record (two weekdays and one weekend day), completed at home and returned at the subsequent visit. Nutrient intakes were analyzed using GENI software (37) and compared with international dietary reference values (38, 39).

Physical activity (PA) was assessed using the validated French version of the International Physical Activity Questionnaire–Short Form (IPAQ-SF) (40). Results expressed in minutes per day were interpreted using standard metabolic equivalent of task (MET) values according to the Compendium of Physical Activities, then classified into vigorous PA (>6 METs), moderate PA (3–6 METs), and walking (3–4 METs). Daily screen time exceeding 2 h (television, computer or smartphone use) was used as an indicator of sedentary behavior, according to World Health Organization recommendations. Total energy expenditure (TEE) was assessed using GENI questionnaire, and GENI software (37).

Quality of life was assessed using two validated questionnaires. CD-specific QoL was assessed using the Arabic version of the CeD Dutch Questionnaire (CDDUX) (41, 42), a 12-item self-administered questionnaire evaluating three domains (communication, having CeD, and diet). Overall scores were interpreted as very poor (0–20), poor (21–40), neutral (41–60), good (61–80), and very good (81–100). Because the questionnaire is self-administered, it was completed only by patients aged ≥9 years after standardized instructions were provided by the study dietitian and in the absence of their parents to encourage independent responses. Generic HRQoL was assessed using the Arabic version of the KIDSCREEN-10 questionnaire (43, 44), a validated 10-item self-administered instrument evaluating physical, psychological, and social well-being. Scores were calculated according to the official KIDSCREEN manual, with higher T-scores indicating better HRQoL. High HRQoL was defined as a T-value ≥ 48.51 on the A7_A-3 Kidscreen-10 scale. This questionnaire was also administered only to patients aged ≥9 years under the same standardized conditions.

### Study design

Following baseline assessments and randomization, participants allocated to the IG attended four standardized workshops (therapeutic education, Mediterranean diet–based nutrition education, culinary workshop, and psychological support), whereas participants allocated to CG attended one therapeutic education workshop only. Each workshop lasted approximately 4 h, comprising two 90-min educational sessions separated by a 30-min refreshment break, followed by a 30–45-min interactive discussion and question-and-answer session. First, both IG and CG groups attended a standardized therapeutic education workshop. Thereafter, patients allocated to the IG attended three additional workshops delivered over three consecutive weeks (one workshop per week), on MD–based nutrition education, practical culinary training, and psychological support.

All sessions were conducted in small groups of 10–15 child–caregiver dyads to facilitate interaction and active participation. Attendance of one caregiver, preferably the family member primarily responsible for grocery shopping and meal preparation, was mandatory. Whenever possible, workshop groups were also organized according to patient age (young children, older children, and adolescents) to facilitate age-appropriate communication, peer interaction and adapt educational activities.

The nutrition education sessions were delivered by a registered dietitian with experience in pediatric nutrition and CeD management. The therapeutic education sessions were supervised by a senior pediatrician specialized in pediatric gastroenterology and CeD. The culinary workshops were conducted by a chief specialized in gluten-free cooking with extensive experience in developing gluten-free recipes. The psychological support sessions were led by a licensed clinical psychologist with experience in pediatric group counseling.

To ensure study fidelity and consistency across study groups and sessions, both intervention and control groups received the same standardized therapeutic education sessions, delivered by the same registered dietitian using identical educational materials and under the supervision of the same pediatrician, who was present throughout each session to answer participants questions and ensure the consistency and accuracy of the information provided. The fidelity of the intervention-specific components was ensured by delivering all workshops according to predefined standardized protocols using identical educational materials, patient handouts, and teaching resources. The same registered dietitian conducted all MD–based nutrition education sessions, the same chief conducted all culinary workshops, and the same licensed clinical psychologist led all psychological support sessions, ensuring that all participants received the same educational content, practical recommendations, and psychological support. In addition, all participants in IG prepared the same standardized recipes during the culinary workshops, ensuring consistency of the practical component of the intervention.

To minimize contamination between study arms, therapeutic education sessions for the IG and CG were conducted separately, and patients from the both groups never attended sessions simultaneously. In addition, all educational sessions were delivered on Saturday in the pediatric department, when no routine outpatient consultations were held. This organization ensured that celiac patients assigned to the IG did not come into contact with those in the CG during their usual clinical follow-up.

Participation in the study was entirely voluntary. No financial compensation was provided to celiac patients or their caregivers. In addition, participation in all educational workshops, pedagogical materials and follow-up support was provided free of charge. No fees were required from participants or their families, in accordance with the study protocol and ethical approval.

The study design and intervention flow are detailed in Supplementary file.

### Standard therapeutic education

The therapeutic education session comprised two complementary modules. The first introduced CeD, covering digestive physiology, disease mechanisms, symptoms, and potential complications. The second focused on the principles of the GFD, including naturally gluten-free foods, foods containing gluten, hidden sources of gluten, food additives, label reading, and the prevention of cross-contamination.

Each session concluded with an interactive question-and-answer discussion jointly led by the dietitian and the pediatrician. To demonstrate the practical feasibility and palatability of a gluten-free diet, celiac patients shared a convivial gluten-free snack accompanied by beverages, savory preparations (mini quiches and mini pizzas), and sweet recipes (brownies, madeleines, and traditional peanut-based cakes prepared without wheat flour). At the end of the therapeutic education session, individual biochemical results obtained at baseline were shared and discussed with each participant under the supervision of the pediatrician. Patients also received an information leaflet summarizing foods allowed and prohibited in a GFD.

### Nutritional intervention

#### MD–based nutrition education

The nutritional education workshop consisted of two complementary sessions. The first session was delivered as an interactive PowerPoint presentation covering the principles of balanced nutrition, energy balance, food groups, the physiological functions of foods and chrononutrition. Particular emphasis was placed on appropriate energy distribution throughout the day, especially breakfast and afternoon snacks. The concepts of glycemic index and glycemic load were also explained. The principles and health benefits of the MD adapted to the GFD were first presented. A specifically designed Mediterranean gluten-free food pyramid illustrated recommended food choices, consumption frequencies, hydration, seasonal foods, and other healthy lifestyle habits. Additional information was provided on nutritional factors influencing vitamin D and iron status, including the role of sunlight exposure in vitamin D synthesis and the interaction between iron and vitamin C in enhancing iron absorption. Recommendations regarding regular physical activity, reduced recreational screen time, and healthy sleep habits were also discussed. The second session aimed to reinforce learning through active participation. Celiac children and adolescents completed interactive educational activities including food card games, quizzes with a point-based scoring system, construction of balanced plates, assembly of Mediterranean dietary pyramids, and practical activities involving coloring and cutting. These activities were designed to strengthen food literacy, facilitate long-term knowledge retention, and improve children’s engagement with healthy eating behaviors.

Each session concluded with an interactive discussion during which CeD patients and caregivers were encouraged to ask questions and discuss practical challenges encountered in the daily management of a GFD. During the refreshment break, Mediterranean-based gluten-free snacks and desserts were served as practical demonstrations of healthy, gluten-free alternatives suitable for everyday consumption.

### Culinary workshop

The culinary workshop consisted of a hands-on practical session jointly supervised by a chef specialized in gluten-free preparations and the registered dietitian. Recipes were selected according to the baseline dietary assessment. Consequently, the workshop focused improving the nutritional quality of commonly consumed gluten-free foods, as well as recipes considered difficult to prepare at home.

Celiac disease patients prepared Mediterranean-style gluten-free bread and pizza with a vegetable-rich sauce containing olive oil, garlic, herbs, to enhance both nutritional quality and palatability. The workshop additionally introduced healthy snack alternatives compatible with a Mediterranean gluten-free dietary pattern, including homemade snacks prepared from dates, nuts, seeds, and unsweetened cocoa. Participants also learned to prepare homemade peanut butter and sesame paste (tahini) as healthier alternatives to commercial chocolate spreads and margarine in sweet preparations.

Throughout the workshop, numerous practical culinary strategies were discussed to improve the nutritional quality of every day meals, increase the use of vegetables, legumes, olive oil, and fish, and enhance children’s acceptance of healthy foods. Practical tips for incorporating vegetables into family meals were also shared. All recipes, menus, and practical cooking recommendations were compiled into a standardized recipe booklet distributed to participating families for home use, a gluten-free food pyramid guide was also provided.

### Psychological support

The psychological component consisted of a single standardized group session led by licensed clinical psychologist. Rather than providing formal psychotherapy or cognitive-behavioral therapy, the session followed a supportive psycho-educational group discussion based on supportive counseling techniques.

The workshop aimed to strengthen self-esteem, facilitate acceptance of the disease and lifelong GFD, improve emotional regulation, and encourage communication between children and their parents. Celiac patients were invited to share personal experiences and discuss common challenges encountered in everyday life, including school meals, family gatherings, eating outside the home, peer relationships, and responding to questions about CeD or dietary restrictions. Sleep habits and recreational screen time were also discussed because of their potential impact on psychological well-being and healthy lifestyle behaviors.

The psychologist facilitated group discussions, encouraged peer support and collaborative problem-solving, and helped celiac patients identify practical coping strategies adapted to their daily lives. Parents were encouraged to exchange experiences and discuss ways to support their children’s dietary adherence while promoting autonomy and positive family communication.

### Outcome assessment

Ongoing standardized follow-up and outcome assessment were ensured in both IG and control CG. At 30 and 60 days after completion of the final workshop, celiac patients attended standardized follow-up visits during which dietary intake and anthropometric measurements were reassessed. Individualized feedback and reinforcement of dietary recommendations were also provided. In addition, between scheduled visits, ongoing dietary support was provided by the study dietitian through weekly telephone calls or WhatsApp messaging. Participants were encouraged to contact the dietitian with practical questions regarding food choices, label reading, recipe preparation, or everyday challenges related to maintaining a GFD. All baseline assessments were repeated at day 90.

### Outcome measures

The primary outcome was the change in MD adherence from baseline to day 90. The secondary outcomes included changes in anthropometric parameters, serum parameters, GFD adherence (CDAT score), dietary intake, PA levels and QoL (CDDUX and KIDSCREEN-10).

### Statistical analysis

Statistical analysis was performed using SPSS version 23. The Shapiro–Wilk test was used to assess the normality of continuous data distribution. Quantitative variables are presented as mean ± standard deviation (SD), whereas qualitative variables are expressed as frequencies (n) and percentages (%). Within-group, comparisons between baseline and day 90 were performed using the paired Student *t*-test for normally distributed variables or the Wilcoxon test for non-normally distributed variables. Inter-group comparisons at day 90 were conducted using the independent Student *t*-test for normally distributed variables or the Mann–Whitney test for non-normally distributed variables. Qualitative variables were analyzed using McNemar test for dichotomous variables or the McNemar–Bowker test for variables with more than two categories for within-group comparisons, and the chi-square (χ^2^) test for inter-group comparisons. A *p*-value < 0.05 was considered statistically significant. To further investigate the longitudinal evolution of the KIDMED score, a linear mixed-effects model was applied, including group (intervention vs. control), time (baseline vs. day 90), and their interaction (group × time) as fixed effects. A random intercept was included to account for baseline inter-individual variability in MD adherence, and an unstructured covariance matrix was chosen to avoid imposing assumptions on the correlation structure between repeated measures, given the short follow-up period and heterogeneous variability between individuals. In addition, a multivariate linear regression analysis was conducted to identify factors associated with changes in MD adherence (ΔMD). The independent variables included in the model were selected based on findings from our previous observational study, which identified baseline dietary behaviors associated with MD in the absence of intervention. The final model included group (intervention vs. control), difficulty in preparing gluten-free bread (yes vs. no), skipping meals (modeled using two dummy variables: yes vs. no, and rarely vs. no) and gluten-free food budget (>5,000 vs. ≤5,000 DZD), and baseline GFD adherence score. The assumptions of linear regression were evaluated by assessing linearity through residuals versus fitted values, independence of errors using the Durbin–Watson statistic, and multicollinearity via variance inflation factors (VIF). No significant violations were detected.

Analyses were conducted on celiac patients with complete follow-up data (per-protocol population). As outcome data were not available for those lost to follow-up, an intention-to-treat analysis was not performed due to missing post-intervention data.

## Results

### Study population characteristics

Baseline characteristics were similar between IG and CG groups, including age (12 ± 4 vs. 11 ± 4 years), sex distribution (57% vs. 71% girls), and duration of GFD adherence (6 ± 4 vs. 6 ± 5 years) (Table 1). A higher proportion of families in the IG reported a gluten-free food budget >5,000 DZD (56% vs. 29%, *p* < 0.05) (Table 1).

**TABLE 1:** Characteristics of celiac pediatric patients.

Parameters	Intervention group	Control group
*N*	35	24
Female n (%)	20 (57)	17 (71)
Mean age (years)	12 ± 4	11 ± 4
Age at onset GFD (years)	7 ± 4	8 ± 4
Duration of GFD (years)	6 ± 4	6 ± 5
Household food budget *n* (%)		
≤21,500 DZD	14 (41)	9 (43)
>21,500 DZD	20 (59)	12 (57)
Gluten-free food budget *n* (%)		
≤5,000 DZD	15 (44)	15 (71)
>5,000 DZD	19 (56)	6 (29)

n, number of patients; DZD, national currency; GFD, gluten-free diet. Results are presented as mean ± standard deviation and percentages for a total of 59 children and adolescents with celiac disease (IG = 35 patients; CG = 24 patients). The sample size varied for socioeconomic status (IG = 34 patients; CG = 21 patients).

### Anthropometric parameters

Over the 90-day follow-up, weight (33.53 ± 13.04 to 37.51 ± 13.32 kg, *p* < 0.001), and height (1.39 ± 0.19 to 1.45 ± 0.17 m, *p* < 0.001) increased significantly in IG, while body mass index (BMI) remained stable. In CG, only weight increased slightly (31.74 ± 11.64 to 32.91 ± 11.61 kg, *p* < 0.05), with no change in height or BMI. The proportion of underweight lowered in IG (46%–32%), whereas it increased in CG (46%–53%), with no significant difference between groups at day 90 (Table 2).

**TABLE 2:** Anthropometric characteristics and serum parameters of celiac intervention group before and after nutritional intervention, compared with celiac control group.

Parameters	Intervention group			Control group			*p*-value Inter-group
	Baseline	Day 90	*p*-value	Baseline	Day 90	*p*-value	
Weight (kg)	33.53 ± 13.04	37.51 ± 13.32	<0.001	31.74 ± 11.64	32.91 ± 11.61	0.011	NS
Height (m)	1.39 ± 0.19	1.45 ± 0.17	<0.001	1.12 ± 0.61	1.33 ± 0.35	NS	NS
BMI (kg/m^2^)	16.54 ± 2.19	17.19 ± 2.5	NS	16.33 ± 2.30	16.39 ± 2.36	NS	NS
Underweight *n* (%)	16 (46)	9 (32)		11 (46)	10 (53)		
Normal weight *n* (%)	18 (51)	18 (64)		12 (50)	8 (42)		
Overweight/Obesity *n* (%)	1 (3)	1 (4)		1 (4)	1 (5)		
Albumin g/L	31.23 ± 12.35	42.99 ± 5.24	0.010	40.10 ± 12.87	32.04 ± 9.97	0.048	NS
Triglyceride g/L	1.21 ± 0.10	1.33 ± 0.06	0.017	1.17 ± 0.07	1.35 ± 0.17	0.026	NS
Total cholesterol g/L	1.63 ± 0.38	1.51 ± 0.23	NS	1.59 ± 0.23	1.62 ± 0.14	NS	NS
Calcium mg/L	82.36 ± 4.83	85.66 ± 8.21	NS	82.71 ± 13.85	86.51 ± 11.21	NS	NS
Phosphorus mg/L	46.04 ± 5.17	45.52 ± 4.94	NS	44.23 ± 4.07	43.03 ± 5.19	NS	NS
Vitamin D ng/mL	18.45 ± 7.20	34.07 ± 8.73	<0.001	13.42 ± 5.37	25.90 ± 9.83	0.001	0.037
Iron μg/mL	0.57 ± 0.24	0.61 ± 0.27	NS	0.80 ± 0.28	0.73 ± 0.10	NS	NS
Ferritinemia μg/L	32.20 ± 21.88	42.30 ± 24.99	NS	38.36 ± 20.33	21.67 ± 18.42	NS	NS
MCV μm^3^	80.47 ± 2.05	81.27 ± 5.89	NS	82.37 ± 8.76	84.57 ± 4.17	NS	NS

n, number of patients; BMI, body mass index (weight/height, kg/m^2^); MCV, mean corpuscular volume; NS, not significant. Results are presented as mean ± standard deviation and percentages for 59 children and adolescents with celiac disease (IG, *n* = 35 patients; CG, *n* = 24 patients) at baseline, and for 47 children and adolescents with celiac disease (IG, *n* = 28 patients; CG, *n* = 19 patients) at day 90 of follow up. For serum parameters, results are presented as mean ± standard deviation for 45 children and adolescents with celiac disease (IG, *n* = 23 patients; CG, *n* = 22 patients) at baseline, and 26 children and adolescents with celiac disease (IG, *n* = 14 patients; CG, *n* = 12 patients) at day 90 of follow up. Statistical significance was defined as *p* ≤ 0.05. The inter-group *p*-value was estimated at day 90 of follow up between intervention and control celiac groups.

### Biological parameters

Albumin concentrations increased in IG (31.23 ± 12.35 to 42.99 ± 5.24 g/L, *p* < 0.05), whereas they decreased in CG (40.10 ± 12.87 to 32.04 ± 9.97 g/L, *p* < 0.05). Triglyceride concentrations increased slightly in both groups. Vitamin D levels improved significantly in both IG (*p* < 0.001) and CG (*p* = 0.001) with a between-group difference at day 90 (*p* < 0.05). All the other parameters remained unchanged (Table 2).

### Adherence to MD and GFD

Mediterranean diet adherence score increased significantly in IG, rising from low adherence levels (2.49 ± 1.88) to moderate-to-high adherence (7.49 ± 2.43; *p* < 0.001). This improvement was accompanied by a marked shift in dietary classification, with a reduction in low adherence from 66% to 9% and an increase in optimal adherence from 0% to 54%. In contrast, only a minimal and borderline improvement was observed in CG (2.17 ± 1.79 to 2.75 ± 1.89; *p* = 0.05), with no celiac patients reaching optimal adherence at follow-up. The between-group difference at day 90 was highly significant (*p* < 0.001). Adherence to GFD improved in IG, with scores decreasing from 11 ± 4 to 6 ± 4, (*p* < 0.001). Poor adherence reduced from 91% to 40%, while excellent adherence increased from 0% to 26%. A smaller change was observed in CG (11 ± 5 to 10 ± 6, *p* < 0.05). The difference between groups was significant at day 90 (*p* < 0.001). Dietary habits related to GFD improved significantly in IG, including reduced consumption of gluten-containing cereals (57%–11%; *p* < 0.001), improved bread preparation (54%–3%; *p* < 0.001), lowered perceived low satiety (37%–6%; *p* = 0.001), and reduced meal distraction (49%–20%; *p* < 0.05). Skipping meals also decreased significantly (*p* = 0.001), with an increase in participants reporting no meal skipping (29%–77%). In contrast, in CG, most outcomes remained stable over time. A significant change was observed only for skipping meals, with an increase in celiac patients reporting no meal skipping (*p* < 0.05). Meal sociability and other dietary difficulties remained largely unchanged. A significant between-group difference at follow-up was observed for eating alone (*p* < 0.05), with higher frequency in CG (Table 3).

**TABLE 3:** Quality of Mediterranean diet and GFD in celiac intervention group before and after nutritional intervention, compared with celiac control group.

Parameters	Intervention group		*p*-value	Control group		*p*-value	*p*-value Inter-group
	Baseline	Day 90		Baseline	Day 90		
Adherence score to MD	2.49 ± 1.88	7.49 ± 2.43	<0.001	2.17 ± 1.79	2.75 ± 1.89	0.05	<0.001
≤3 Low adherence *n* (%)	23 (66)	3 (9)		18 (75)	13 (54)		
4–7 moderate adherence *n* (%)	12 (34)	13 (37)		6 (25)	11 (46)		
≥8 Optimal adherence *n* (%)	0 (0)	19 (54)		0 (0)	0 (0)		
Adherence score to GFD	11 ± 4	6 ± 4	<0.001	11 ± 5	10 ± 6	0.035	0.002
≤2: excellent adherence *n* (%)	0 (0)	9 (26)		0 (0)	1 (4)		
3–6: moderate adherence *n* (%)	3 (9)	12 (34)		7 (29)	8 (33)		
≥7: low adherence *n* (%)	32 (91)	14 (40)		17 (71)	15 (63)		
Dietary habits in GFD n (%)							
Different meal menu for patient	3 (9)	1 (3)	NS	5 (21)	4 (17)	NS	NS
Consumption of gluten-containing cereals	20 (57)	4 (11)	<0.001	17 (71)	17 (71)	NS	<0.001
Difficulty during GFD n (%)							
Complicated bread preparation	19 (54)	1 (3)	<0.001	14 (58)	13 (54)	NS	<0.001
Tasteless gluten-free products	13 (37)	7 (20)	NS	9 (38)	8 (33)	NS	NS
Difficulty eating out	29 (83)	23 (66)	NS	12 (50)	11 (46)	NS	NS
Low satiety of gluten-free foods	13 (37)	2 (6)	0.001	10 (42)	10 (42)	NS	0.001
Meal sociability n (%)							
Alone	5 (14)	(3) 1	NS	3 (13)	7 (29)	NS	0.004
With family	20 (57)	24 (69)	NS	13 (54)	15 (63)	NS	NS
Alone + with family	10 (29)	10 (29)	NS	8 (33)	7 (29)	NS	NS
Distraction during meals *n* (%)	17 (49)	7 (20)	0.006	12 (50)	9 (38)	NS	NS
Skipping meals *n* (%)			0.001			NS	0.033
No	10 (29)	27 (77)		11 (46)	12 (50)		
Yes	14 (40)	2 (6)		10 (42)	7 (29)		
Rarely	11 (31)	6 (17)		3 (13)	5 (21)		

n, number of patients; GFD, gluten-free diet; NS, not significant. Results are presented as mean ± standard deviation and percentages for 59 children and adolescents with celiac disease (IG, *n* = 35; CG, *n* = 24 patients). Statistical significance was defined as *p* ≤ 0.05. The inter-group *p*-value was estimated at day 90 of follow up between intervention and control celiac groups.

### Nutritional intake

Although there was no difference in total energy intake (TEI) in IG, protein intake increased significantly from 11% TEI to 14% TEI (*p* < 0.001), including vegetable proteins (8%–10% TEI; *p* < 0.05), and animal proteins (3%–4% TEI; *p* < 0.05). A decrease in animal proteins percentage was observed in CG (5%–3%; *p* < 0.05), with a between-group difference for total protein (*p* < 0.05) and animal proteins (*p* = 0.05). Total carbohydrate intake increased in CG (57%–60% TEI; *p* < 0.05), but remained stable in IG (56%–54% TEI) at day 90, with a significant inter-group difference (*p* = 0.001). Qualitatively, complex carbohydrates showed a significant inter-group difference at day 90 (IG: 39%–36% vs. CG: 41%–43% TEI; *p* < 0.05). Total lipid intake decreased in the CG (31%–28% of TEI; *p* < 0.05) but remained stable in the IG (32% of TEI), resulting in a significant between-group difference at day 90 (*p* < 0.05). Similarly, PUFA intake decreased in the CG (10%–8% of TEI; *p* < 0.05), with significant between-group differences for PUFA (*p* < 0.05), MUFA (*p* < 0.05), and α-linolenic acid (*p* = 0.05) at day 90. Cholesterol intake increased significantly in the IG (234.46 ± 141.37 to 374.67 ± 223.89 mg/day; *p* < 0.05), whereas no significant change was observed in the CG. In the IG, dietary fiber intake increased from 13.8 ± 4.17 to 19.48 ± 6.88 g/day (*p* < 0.001), with a significant between-group difference at day 90 (*p* < 0.05). Iron (6.24 ± 2.20 to 7.75 ± 2.72 mg/day; *p* < 0.05), calcium (452.34 ± 168.80 to 662.47 ± 213.24 mg/day; *p* < 0.001), and vitamin C (47.89 ± 21.92 to 65.07 ± 41.36 mg/day; *p* < 0.05) intakes also increased significantly in the IG. Significant between-group differences at day 90 were observed for calcium (*p* < 0.001) and vitamin C (*p* = 0.05) (Table 4).

**TABLE 4:** Dietary intake in celiac intervention group before and after intervention compared to celiac control group, Mediterranean diet score and DRIs.

Parameters	Intervention group			Control group			*p*-value Inter-group	DRIs
	Baseline	Day 90	*p*-value	Baseline	Day 90	*p*-value		
TEI (Kcal/day)	1,737.92 ± 487.3	1,809.85 ± 479.81	NS	1,672.3 ± 415.72	1,713.57 ± 353.75	NS	NS	–
Total proteins g/day (% TEI)	49.77 ± 19.15 (11)	62.62 ± 20.61 (14)	<0.001	51.62 ± 18.89 (12)	50.01 ± 16.78 (12)	NS	0.009	10–15^a^
Vegetable proteins g/day (% TEI)	35.38 ± 16.89 (8)	43.86 ± 18.51 (10)	0.004	32.57 ± 13.07 (8)	37.05 ± 14.06 (9)	NS	NS	6–9^a^
Animal proteins g/day (% TEI)	14.39 ± 7.68 (3)	18.76 ± 11.76 (4)	0.026	19.06 ± 11.55 (5)	12.97 ± 6.8 (3)	0.005	0.052	4–6^a^
Total carbohydrates g/day (% TEI)	240.17 ± 58.97 (56)	244.25 ± 67.28 (54)	NS	236.59 ± 65.78 (57)	255.51 ± 54.05 (60)	0.030	0.001	45–60^b^
Simple carbohydrates g/day (% TEI)	75.29 ± 30.84 (17)	81.63 ± 30.89 (18)	NS	66.61 ± 26.44 (16)	72.53 ± 30.13 (17)	NS	NS	10^a^
Complex carbohydrates g/day (% TEI)	171.13 ± 53.57 (39)	162.61 ± 57.46 (36)	NS	174.3 ± 55.61 (41)	187.30 ± 48.66 (43)	NS	0.002	40–45^a^
Total lipids g/day (% TEI)	64.24 ± 26.77 (32)	64.71 ± 23.4 (32)	NS	57.73 ± 16.46 (31)	54.61 ± 15.97 (28)	0.036	0.042	20–35^b^
Saturated FA g/day (% TEI)	17.04 ± 6.73 (9)	16.48 ± 6.5 (8)	NS	16.54 ± 5.82 (9)	16.87 ± 5.22 (9)	NS	NS	<10^a^
MUFA g/day (% TEI)	18.45 ± 8.34 (9)	20.53 ± 9.03 (10)	NS	16.79 ± 5.42 (9)	16.63 ± 5.64 (9)	NS	0.049	10–20^a^
PUFA g/day (% TEI)	22.01 ± 11.64 (11)	20.79 ± 10.83 (10)	NS	18.35 ± 6.9 (10)	14.8 ± 7.45 (8)	0.019	0.025	5–10^a^
Linoleic acid (% TEI)	8	8	NS	9	6	0.037	NS	4–10^b^
Alpha-LA (% TEI)	1	0.9	NS	1.2	0.6	0.005	0.054	0.5–1^b^
Cholesterol mg/day	234.46 ± 141.37	374.67 ± 223.89	0.002	287.91	337.14 ± 235.65	NS	NS	<300^b^
Fiber g/day	13.8 ± 4.17	19.48 ± 6.88	<0.001	14.09 ± 6.41	14.92 ± 5.63	NS	0.01	17–19^b^
Iron mg/day	6.24 ± 2.2	7.75 ± 2.72	0.002	7.04 ± 2.82	6.83 ± 2.53	NS	NS	11–13^b^
Calcium mg/day	452.34 ± 168.8	662.47 ± 213.24	<0.001	452.58 ± 174.11	466.16 ± 139.33	NS	<0.001	800–1,150^b^
Vitamin D μg/day	2.42 ± 2.28	3.43 ± 2.97	0.115	2.52 ± 1.4	2.54 ± 1.32	NS	NS	15^b^
Vitamin C mg/day	47.89 ± 21.92	65.07 ± 41.36	0.024	43.67 ± 16.51	46.03 ± 25.08	NS	0.053	70–100^b^

DRI, dietary recommended intake; TEI, total energy intake; MUFA, monounsaturated fatty acids; PUFA, polyunsaturated fatty acids; LA, linolenic acid. Results are presented as mean ± standard deviation of 59 celiac children and adolescent aged 5–18 years (IG = 35, CG = 24). ^a^Martin (38); ^b^EFSA (39). DRIs of each macro-nutrient are presented in recommended % of total energy intake; DRIs of food groups consumption are presented in portions. Significance was determined using Pearson test. Statistical significance was considered for *p* ≤ 0.05. NS, not significant. The inter-group *p*-value was estimated at day 90 of follow up between intervention and control celiac groups.

### PA, TEE and sedentary behavior

Physical activity levels remained stable in both groups. However, sedentary time decreased slightly in IG (3.3 ± 1.2 to 3.0 ± 1.0 h/day; *p* < 0.05), with no change in CG. In addition, a significant between-group difference in TEE was observed at day 90, with higher values in IG group (*p* = 0.001) (Table 5).

**TABLE 5:** Energy expenditure and quality of life in celiac intervention group before and after nutritional intervention, compared with celiac control group.

Parameters	Intervention group			Control group			*p*-value Inter-group
	Baseline	Day 90	*p*-value	Baseline	Day 90	*p-*value	
MET> 6: vigorous (min/day)	42.66 ± 38.96	45.2 ± 39.61	NS	34.89 ± 28.64	34.66 ± 28.71	NS	NS
(*n*) min-max	(33) 10–140	(35) 10–140		(20) 10–100	(21) 10–120		
MET 3-6: moderate (min/day)	62.58 ± 40.06	58.29 ± 35.91	NS	51.00 ± 37.19	47.86 ± 27.9	NS	NS
(*n*) min-max	(33) 10–180	(35) 20–180		(20) 10–180	(21) 10–120		
MET sedentary (h/day)	3.3 ± 1.2	3 ± 1	0.031	4.1 ± 2	4.2 ± 2	NS	0.005
(*n*) min-max	(33) 1–6	(35) 1–6		(20) 2–10	(21) 1–10		
Total energy expenditure (Kcal/day)	1,831.83 ± 488.24	1,961.13 ± 493.47	0.001	1,718.05 ± 397.99	1,782.86 ± 231.88	NS	NS
Quality of life							
CD-DUX (score)	41.54 ± 8.46	50.58 ± 10.04	<0.001	40.88 ± 9.49	45.78 ± 13.22	0.053	NS
Communication about CD	50.12 ± 16.68	55.64 ± 15.54	0.03	49.02 ± 12.68	55.29 ± 18.67	NS	NS
Having celiac disease	39.51 ± 12.11	47.18 ± 9.78	<0.001	38.04 ± 16.12	44.58 ± 19.88	0.053	NS
GFD	38.27 ± 8.69	48.97 ± 12.36	<0.001	38.24 ± 9.44	41.18 ± 12.19	NS	0.048
Kidscreen-10 (T-value)	45.89 ± 5.47	51.46 ± 7.52	<0.001	45.29 ± 7.11	46.58 ± 7.80	NS	0.053

MET, metabolic equivalent of task; n, number of participants; min–max: minimum–maximum; NS, not significant. Results are presented as mean ± standard deviation for 59 children and adolescents with celiac disease (IG, *n* = 35 patients; CG, *n* = 24 patients). Statistical significance was defined as *p* ≤ 0.05. The inter-group *p*-value was estimated at day 90 of follow up between intervention and control celiac groups.

### QoL (CD-DUX and KIDSCREEN-10)

The CD-DUX score increased from 41.54 ± 8.46 at day 0 to 50.58 ± 10.04 at day 90 (*p* < 0.001) in IG, and from 40.88 ± 9.49 to 45.78 ± 13.22 (*p* = NS) in CG, with no significant inter-group difference. Regarding the “communication about CD” domain, scores rose from 50.12 ± 16.68 to 55.64 ± 15.54 in IG (*p* < 0.05). For the “having CeD” domain, scores increased from 39.51 ± 12.11 to 47.18 ± 9.78 in IG (*p* < 0.001), and from 38.04 ± 16.12 to 44.58 ± 19.88 in CG (*p* = NS). The GFD-related domain improved from 38.27 ± 8.69 to 48.97 ± 12.36 in IG (*p* < 0.001), whereas no significant increase was noted in CG, but with a significant inter-group difference at day 90 (*p* < 0.05). The Kidscreen-10 T-value enhanced from 45.89 ± 5.47 to 51.46 ± 7.52 in IG (*p* < 0.001), with significant between-group difference (*p* = 0.05) (Table 5).

### Factors associated with changes in MD adherence

The linear mixed model analysis showed no significant main effect of group on MD adherence (β = −0.32; 95% CI [−1.30; 0.66], *p* = NS) nor of time (β = −0.58; 95% CI [−1.51; 0.34], *p* = NS) (Table 6). However, a significant group × time interaction was observed (β = −4.42; 95% CI [−5.61; −3.22], *p* < 0.001), indicating that changes in MD adherence over time differed significantly between IG and CG. Multivariate linear regression analysis showed that group allocation was the strongest predictor of improved MD adherence (β = 4.448; *p* < 0.001). Difficulty preparing gluten-free bread was also significantly associated with greater improvement (β = 1.518; *p* < 0.05). Skipping meals was not significantly associated with ΔMD (yes vs. no: β = −1.291; *p* = NS; rarely vs. no: β = −0.420; *p* = NS), nor was baseline GFD adherence (β = −0.040; *p* = NS). Household gluten-free food budget was not a significant predictor (β = 0.451; *p* = NS) (Table 7).

**TABLE 6:** Linear mixed model analysis of MD according to group, time, and their interaction.

	β (Estimate)	95% CI	*p*-value
Groupe (control vs intervention)	−0.32	[−1.30; 0.66]	NS
Temps (baseline vs. day 90)	−0.58	[−1.51; 0.34]	NS
Groupe × Temps	−4.42	[−5.61; −3.22]	<0.001

A linear mixed-effects model with a random intercept for subjects was used. Results are presented as β coefficients with 95% confidence intervals (CI) and *p*-values for fixed effects (group, time, and group × time interaction). An unstructured covariance matrix was used to model repeated measures. Statistical significance was set at *p* < 0.05. The analysis included 59 participants (intervention: *n* = 35; control: *n* = 24). NS, not significant.

**TABLE 7:** Multivariate linear regression analysis identifying factors associated with changes in Mediterranean diet adherence score (ΔMD).

Variable	β	95% CI	p-value
Group (intervention vs. control)	4.448	[3.245; 5.652]	<0.001
Difficulty preparing gluten-free bread (yes vs. no)	1.518	[0.257; 2.779]	0.019
Skipping meals (yes vs. no)	−1.291	[−2.719; 0.137]	NS
Skipping meals (rarely vs. no)	−0.420	[−1.972; 1.131]	NS
Gluten-free diet adherence score	−0.040	[−0.170; 0.089]	NS
Household gluten-free food budget (>5,000 vs. ≤5,000 DZD)	0.451	[−0.772; 1.675]	NS

Multiple linear regression analysis was performed to identify predictors of change in Mediterranean diet adherence score (ΔMD). Results are reported as β coefficients with 95% confidence intervals (CI) and *p*-values. The model included group, baseline dietary behaviors, GFD adherence score, and household gluten-free budget. The multivariate linear regression model was statistically significant (F_(6,52)_ = 11.62, *p* < 0.001), explaining 57.3% of the variance in ΔMD (R^2^ = 0.573; adjusted R^2^ = 0.523). The Durbin–Watson statistic (1.981) indicated no autocorrelation of residuals. Multicollinearity diagnostics showed acceptable values (VIF < 2 for all variables). A *p*-value < 0.05 was considered statistically significant. The analysis included *n* = 59 participants (intervention: *n* = 35; control: *n* = 24).

## Discussion

The present randomized controlled trial evaluated the effectiveness of a multidisciplinary intervention combining MD-based nutrition education, culinary workshops, and group psychological support in children and adolescents with CeD. Compared with standard care, the intervention improved significantly adherence to both GFD and MD. It also enhanced dietary quality, reduced sedentary time, improved GFD-related QoL, and was associated with higher serum vitamin D concentrations. These findings suggested that nutrition education, culinary workshops, and group psychological support provided important benefits beyond conventional dietary counseling.

One of the main findings of this study was the marked improvement in adherence to both GFD and MD following the multidisciplinary intervention. Celiac children and adolescents in the IG achieved higher GFD adherence scores, lower gluten exposure, and reported fewer practical barriers to maintaining the diet, particularly regarding gluten-free bread preparation and the low satiety of gluten-free products. Adherence to MD also promoted substantially, with more than half of IG celiac patients achieving optimal KIDMED scores after 90 days, whereas only modest changes were observed in CG. These findings are in line with previous data showing that structured dietitian-led educational interventions enhanced both GFD adherence and dietary quality in children with CeD (1, 31, 45, 46). Practical educational strategies, such as food label reading, meal planning, food selection, and problem-solving have also been identified as key determinants of successful long-term GFD management (23, 30, 47–50). Furthermore, although evidence remains limited in pediatric CeD, culinary education has similarly improved food literacy and MD adherence in non-celiac pediatric populations (51, 52).

Another important component of our intervention was the inclusion of a brief group-based psychological workshop designed to promote self-esteem, encourage peer discussion, strengthen parent–child communication, and develop practical coping strategies for common social situations, including school meals and family gatherings. Although the independent contribution of this component cannot be isolated within our study design, it likely helped reduce emotional and social barriers that interfere with long-term dietary adherence. This explanation is supported by qualitative evidence indicating that children and adolescents with CeD frequently experience anxiety related to school meals and the daily management of the GFD, whereas social eating often results in emotional distress and the need to justify dietary restrictions (53). In addition, parental attitudes toward the disease are important determinants of children’s psychological adjustment (54). Furthermore, this psychosocial approach is also consistent with the broader Mediterranean lifestyle, which emphasizes shared meals, family interaction, and healthy living beyond food choices alone (55).

Our results showed that the IG group had a higher baseline budget allocated to gluten-free foods despite randomization. Although both study groups received identical recommendations promoting affordable, naturally gluten-free foods, this baseline imbalance should be considered when interpreting the observed improvement in GFD adherence. Greater financial resources may have facilitated access to commercial gluten-free products, particularly gluten-free flour. This issue is especially relevant in the Maghreb, where bread is a staple food, and gluten-free bread and flour remain relatively expensive (56). In this context, gluten-specific food insecurity has been identified as one of the leading causes of poor adherence to the gluten-free diet (15, 21). Likewise, neighborhood socioeconomic deprivation has been associated with poorer clinical outcomes in pediatric celiac disease, highlighting the potential impact of socioeconomic barriers on disease management (57). These challenges are further compounded by the high cost and increasing availability of ultra-processed gluten-free products, which may compromise both affordability and dietary quality (58, 59).

Beyond improving GFD and MD adherence, this multidisciplinary intervention based on the MD also enhanced several indicators of dietary quality. Compared with the CG, participants in the intervention arm achieved significantly higher intakes of total protein, dietary fiber, calcium, and a more favorable fatty acid profile after 90 days follow-up. These findings align with previous intervention studies showing that individualized nutritional counseling strengthened the overall nutritional quality of the diet in children with CeD compared with routine follow-up (31, 45, 60, 61). Similarly, increasing the consumption of naturally nutrient-dense gluten-free foods, including legumes, vegetables, fruits, nuts, and pseudo-cereals, has been shown to improve dietary fiber intake, lower the dietary glycemic index, and enhance micronutrient intake in pediatric CeD (1, 3, 8, 23, 29). However, our results showed that calcium, iron, vitamin C, and particularly vitamin D intakes remained below age-specific recommendations in both groups. Our findings support previous pediatric studies showing that strict adherence to GFD does not necessarily ensure nutritional adequacy (14, 45, 62, 63). A recent systematic review by Russell et al. further confirmed that micronutrient deficiencies remain common among individuals following a GFD, and suggested that these inadequacies may be attributable to the nutritional characteristics of the diet itself rather than to persistent villous atrophy (2). Moreover, Muhammad et al. reported that improvements in nutritional status became more evident during prolonged follow-up (48). These findings suggest that the 90-days duration of our intervention may have been insufficient to achieve the full nutritional benefits of dietary rehabilitation in IG. In this context, the persistence of vitamin D and iron inadequacies underscores the importance of continued nutritional monitoring, individualized dietary counseling, and appropriate supplementation when clinically indicated throughout childhood and adolescence (7, 11, 14, 38, 64, 65).

Interestingly, our results showed that although dietary vitamin D intake remained well below recommendations, and did not significantly increase during the intervention, serum vitamin D concentrations increased significantly within both groups, and were significantly higher in IG than in CG, at day 90. This discrepancy suggests that improved vitamin D status was probably influenced by factors beyond dietary intake, including seasonal variation, sunlight exposure and progressive intestinal recovery following continued adherence to GFD (65). Consequently, serum vitamin D concentrations should not be considered as direct marker of dietary improvement alone.

In our study, the intervention did not significantly increase moderate-to-vigorous PA; however, sedentary time decreased significantly compared with standard care, representing a clinically meaningful behavioral change. This finding is clinically relevant, as excessive recreational screen time in previous studies has been associated with overweight, sleep disturbances, and depressive symptoms in adolescents (66). Moreover, regular PA combined with adherence to the MD has been associated with better bone health in pediatric CeD (67). Future multidisciplinary interventions should therefore incorporate structured strategies to promote regular PA alongside nutritional education.

Quality of life also improved following the intervention. Compared with CG celiac patients, IG group showed significant greater improvement in GFD dimension of the CD-DUX questionnaire. This might be attributed to the better GFD adherence but also to the multidisciplinary interventional approach. Within the IG, significant improvements were additionally observed in CeD related QoL, as well as HRQoL, suggesting favorable effects on both disease-specific and general well-being. These findings are consistent with previous studies demonstrating that better adherence to GFD and healthier dietary patterns are associated with improved QoL in pediatric CeD (23, 24, 68). Beyond nutritional improvements, the intervention may have eased the daily challenges of gluten-free dietary management, contributing to better adherence, greater confidence, and improved QoL (47, 69). Nevertheless, these findings should be interpreted cautiously because QoL in CeD is a dynamic construct influenced by biological, psychological and dietary related factors, as well as social and family context (68).

Multivariate analyses confirmed that improvement in MD adherence remained independently associated with participation in the multidisciplinary MD-based intervention after adjustment for baseline characteristics. Although baseline difficulty in preparing gluten-free bread predicted greater improvement in MD adherence, this finding contrasts with our previous observational study (21), in which it was associated with poorer adherence in the absence of an intervention. Taken together, these findings suggest that the educational program helped participants overcome practical barriers by strengthening food literacy rather than simply increasing nutrition knowledge. Indeed, nutrition knowledge alone does not necessarily translate into behavioral change, as its implementation is constrained by environmental, cultural, and individual factors (70, 71). In this context, Silva et al. proposed that improving MD adherence requires an integrated educational approach, combining nutrition education, culinary skills, psychology, family engagement, and experiential learning (72). Moreover, although in previous studies, household income has been associated with diet quality in children with CeD (73–75), our multivariable analysis showed that improvement in MD adherence was associated with the intervention independently of socioeconomic variables considered.

Importantly, this improvement in MD adherence remained independently associated with the intervention after adjustment for the household gluten-free food budget. The household budget allocated to gluten-free foods was not an independent predictor of improvement in the KIDMED score. In addition, no significant between-group differences were observed in the overall household food budget. Together, these findings suggest that the beneficial effects of the intervention were unlikely to be explained solely by greater financial resources. Instead, they may reflect the emphasis placed on affordable, seasonal, and locally available Mediterranean foods, together with improved food literacy and dietary management. This is supported by Baltaci et al., who showed that strengthening parental cooking self-efficacy can improve dietary quality even in economically vulnerable families (76). Given that commercially available gluten-free products are substantially more expensive and often nutritionally inferior to their conventional counterparts (77, 78), promoting naturally MD based-gluten-free foods may represent both a nutritionally and economically sustainable strategy for the long-term management of pediatric CeD.

Finally, several limitations should be considered when interpreting our findings. First, increased awareness and motivation related to participation in a clinical trial may have influenced celiac patients behaviors (Hawthorne effect). Second, the 90-day follow-up was too short to assess the long-term sustainability of the intervention. Third, the relatively small sample size may have limited statistical power for some secondary outcomes. Attrition was higher in the CG than in the IG despite baseline randomization. This differential loss to follow-up may have introduced attrition bias analysis by preferentially retaining participants who remained available for follow-up in the study. Also, the English versions of CDAT and KIDMED questionnaires were translated and culturally adapted by the research team without undergoing formal cross-cultural validation. Although care was taken to preserve semantic and cultural equivalence, the absence of formal validation may have affected their psychometric properties. Future studies should formally validate these translated versions prior to their wider use. Dietary intake was assessed using self-reported food records and was therefore subject to recall and reporting bias. In addition, because the intervention combined nutritional education, culinary workshops, and psychological support, the individual contribution of each component could not be determined. Another limitation is that the baseline imbalance in the household budget allocated to gluten-free foods between groups should also be considered when interpreting the findings, although this variable was not independently associated with improvement in MD adherence in the multivariable analysis. Future studies using larger samples, longer follow-up, and factorial designs would help confirm these findings and clarify the contribution of each intervention component.

Despite these limitations, to our knowledge, this study is among the first randomized controlled trials evaluating a multidisciplinary intervention integrating MD–based education, culinary training, and psychosocial support in children and adolescents with CeD. These findings support the integration of multidisciplinary educational programs into routine pediatric celiac care and provide a strong rationale for larger multicenter trials with longer follow-up to confirm their effectiveness and long-term sustainability.

## Conclusion

The MD-based intervention in pediatric celiac population induces an increase in adherence to MD and GFD. These changes are linked to healthier eating behaviors. In addition, the intervention promotes favorable growth outcomes, although, no difference is noted in PA parameters. In contrast, the CG shows only minimal or inconsistent improvements across these parameters. Our findings highlight that effective dietary management of pediatric CeD requires psychological and MD based dietary approach alongside with continuous support, and consequently, supports the integration of multidisciplinary approaches into standard care. Further large-scale and long-term studies are required.

## Data Availability

The raw data supporting the conclusions of this article will be made available by the authors, without undue reservation.

## References

[1] Jiang Z Gidrewicz D Chen M Wu J Nasser R Hammond CB et al. A gluten-free food guide used in diet education to improve diet quality in children with newly diagnosed celiac disease: a pilot randomized controlled trial. *Br J Nutr.* (2025) 134:991–1002. 10.1017/S0007114525105618 41190444

[2] Russell LA Alliston P Armstrong D Verdu EF Moayyedi P Pinto-Sanchez MI. Micronutrient deficiencies associated with a gluten-free diet in patients with celiac disease and non-celiac gluten or wheat sensitivity: a systematic review and meta-analysis. *J Clin Med.* (2025) 14:4848. 10.3390/jcm14144848 40725540 PMC12296119

[3] Abdi F Zuberi S Blom J Armstrong D Pinto-Sanchez M. Nutritional considerations in celiac disease and non-celiac gluten/wheat sensitivity. *Nutrients.* (2023) 15:1475. 10.3390/nu15061475 36986205 PMC10058476

[4] Cardo A Churruca I Lasa A Navarro V Vázquez-Polo M Pérez-Junkera G et al. Nutritional imbalances in adult celiac patients following a gluten-free diet. *Nutrients.* (2021) 13:2877. 10.3390/nu13082877 34445038 PMC8398893

[5] Mazzola A Zammarchi I Valerii M Spisni E Saracino I Lanzarotto F et al. Gluten-free diet and other celiac disease therapies: current understanding and emerging strategies. *Nutrients.* (2024) 16:1006. 10.3390/nu16071006 38613039 PMC11013189

[6] Luque V Crespo-Escobar P Hård Af Segerstad EM Koltai T Norsa L Roman E et al. Gluten-free diet for pediatric patients with coeliac disease: a position paper from the ESPGHAN gastroenterology committee, special interest group in coeliac disease. *J Pediatr Gastroenterol Nutr*. (2024) 78:973–95. 10.1002/jpn3.12079 38291739

[7] Simón E Molero-Luis M Fueyo-Díaz R Costas-Batlle C Crespo-Escobar P Montoro-Huguet MA. The gluten-free diet for celiac disease: critical insights to better understand clinical outcomes. *Nutrients.* (2023) 15:4013. 10.3390/nu15184013 37764795 PMC10537989

[8] Caeiro C Pragosa C Cruz M Pereira C Pereira S. The role of pseudocereals in celiac disease: reducing nutritional deficiencies to improve well-being and health. *J Nutr Metab.* (2022) 9:8502169. 10.1155/2022/8502169 35186332 PMC8850039

[9] Dargenio VN Sgarro N La Grasta G Begucci M Castellaneta SP Dargenio C et al. Celiac disease as a model of intestinal malnutrition: mechanisms and nutritional management. *Nutrients.* (2025) 17:3741. 10.3390/nu17233741 41374032 PMC12694320

[10] Jivraj A Hutchinson J Ching E Marwaha A Verdu E Armstrong D et al. Micronutrient deficiencies are frequent in adult patients with and without celiac disease on a gluten-free diet. *Nutrition.* (2022) 103:111809. 10.1016/j.nut.2022.111809 36096056

[11] Mędza A Szlagatys-Sidorkiewicz A. Nutritional status and metabolism in celiac disease: narrative review. *J Clin Med.* (2023) 12:5107. 10.3390/jcm12155107 37568509 PMC10419423

[12] McGrogan L Mackinder M Stefanowicz F Aroutiounova M Catchpole A Wadsworth J. Micronutrient deficiencies in children with coeliac disease; a double-edged sword of both untreated disease and treatment with gluten-free diet. *Clin Nutr.* (2021) 40:2784–90. 10.1016/j.clnu.2020.11.035 33933744

[13] Gessaroli M Frazzoni L Sikandar U Bronzetti G Pession A Zagari RM et al. Nutrient intakes in coeliac disease patients on gluten-free diet. *Eur J Clin Nutr.* (2023) 77:784–93. 10.1038/s41430-023-01280-0 36859658

[14] Papoutsaki M Katsagoni C Papadopoulou A. Nutritional status in children with celiac disease following a gluten-free diet. *Nutrients.* (2025) 17:487. 10.3390/nu17030487 39940345 PMC11820229

[15] Ballestero-Fernández C Varela-Moreiras G Úbeda N Alonso-Aperte E. Nutritional status in Spanish adults with celiac disease following a long-term gluten-free diet is similar to non-celiac. *Nutrients.* (2021) 13:1626. 10.3390/nu13051626 34066195 PMC8151936

[16] Valitutti F Iorfida D Anania C Trovato CM Montuori M Cucchiara S et al. Cereal consumption among subjects with celiac disease: a snapshot for nutritional considerations. *Nutrients.* (2017) 9:396. 10.3390/nu9040396 28420202 PMC5409735

[17] Krueger A Sahay RD Gecili E Henize AW Beck AF Klein M et al. The prevalence and impact of gluten-free food insecurity in pediatric celiac disease. *J Pediatr Gastroenterol Nutr*. (2026) 82:108–17. 10.1002/jpn3.70235 41104661 PMC12780476

[18] Lee AR Ng DL Zong G Green PH. The cost of a gluten-free diet. *Nutr Res.* (2019) 62:17–22. 10.1016/j.nutres.2018.11.002 30803509

[19] Wang X Anders S Jiang Z Bruce M Gidrewicz D Marcon M et al. Food insecurity impacts diet quality and adherence to the gluten-free diet in youth with celiac disease. *J Pediatr Gastroenterol Nutr*. (2024) 79:1180–91. 10.1002/jpn3.12398 39467010 PMC11615129

[20] Aspasia S Emmanuela-Kalliopi K Nikolaos T Eirini S Ioannis S Anastasia M. The gluten-free diet challenge in adults with coeliac disease. *PEC Innov.* (2022) 1:100037. 10.1016/j.pecinn.2022.100037 37213748 PMC10194373

[21] Kaddour FF Mahdad N Bouziane Nedjadi K Latroch C Raiah M Bouchenak M. Quality of life, eating habits and Mediterranean diet in celiac pediatric population. *Med J Nutr Metab*. (2026) 19:68–80. 10.1177/1973798X251383133

[22] Mosher TL Su LJ López-Rivera JA Verma R Keenan K Jericho HA. Longitudinal study of adolescent mental health and quality of life as predictors of college physical health, mental health, and gluten-free diet adherence in celiac disease. *Nutrients.* (2025) 17:3568. 10.3390/nu17223568 41305620 PMC12655353

[23] Rinninella E Cintoni M Raoul P Triarico S Dionisi T Gasbarrini GB et al. The healthy gluten-free diet. *Gastroenterol Insights.* (2021) 12:166–82. 10.3390/gastroent12020015

[24] Psylinakis E Thalassinos N Dafouli AM Kanaki M Manidis A Markaki AG et al. Mediterranean diet and health-related quality of life in patients with celiac disease. *Gastrointestinal Disorders*. (2025) 7:43. 10.3390/gidisord7030043

[25] Bascuñán KA Vespa MC Araya M. Celiac disease: understanding the gluten-free diet. *Nutrients.* (2020) 12:3802. 10.3390/nu12123802 27334430

[26] VeroneseN GianfrediV VolpeM ZanettiM OnderG SilanoMet al. 2025 national guidelines on the Mediterranean diet: executive summary of a joint report by Italian scientific societies and the National Institute of Health task force on clinical practice guidelines. *Nutr Rev*. (2026) nuaf263. 10.1093/nutrit/nuaf263 [Epub ahead of print].41493438

[27] Vici G Malandrino L Giustozzi D Camilletti D Zufolino S Polzonetti V. Assessment of adherence to the Mediterranean diet and physical activity levels in a group of Italian celiac disease patients. *Proceedings*. (2023) 91:232. 10.3390/proceedings2023091232

[28] Peresztegi MZ Szakács Z Vereczkei Z Dakó E Dakó S Lada S et al. Mediterranean diet adherence in celiac patients. *Nutrients.* (2025) 17:788. 10.3390/nu17050788 40077658 PMC11901495

[29] Mager D Cyrkot S Lirette C Brill H Dowhaniuk J Mileski H et al. Nutritional considerations of a paediatric gluten-free food guide. *Br J Nutr.* (2022) 127:421–30. 10.1017/S0007114521000994 33745459

[30] Pohoreski K Horwitz SL Gidrewicz D. Gluten-free diet knowledge and adherence in adolescents with celiac disease: a cross-sectional study. *JPGN Rep*. (2023) 4:e330. 10.1097/PG9.0000000000000330 37600611 PMC10435025

[31] Suárez-González M García CB Treviño SJ Cabo TI Martín JJD. Influence of nutrition education in paediatric coeliac disease: impact of the role of the registered dietitian: a prospective, single-arm intervention study. *J Hum Nutr Diet.* (2020) 33:775–85. 10.1111/jhn.12800 32790023

[32] Husby S Koletzko S Korponay-Szabó I Kurppa K Mearin ML Ribes-Koninckx C et al. ESPGHAN guidelines for diagnosing coeliac disease. *J Pediatr Gastroenterol Nutr.* (2020) 70:141–56. 10.1097/MPG.0000000000002497 31568151

[33] Leffler DA Dennis M Edwards George JB Jamma S Magge S Cook EF et al. A simple validated gluten-free diet adherence survey for adults with celiac disease. *Clin Gastroenterol Hepatol.* (2009) 7:530–6. 10.1016/j.cgh.2008.12.032 19268725

[34] Pedoto D Troncone R Massitti M Greco L Auricchio R. Adherence to gluten-free diet in coeliac paediatric patients assessed through a questionnaire positively influences growth and quality of life. *Nutrients.* (2020) 12:3802. 10.3390/nu12123802 33322343 PMC7764452

[35] Serra-Majem L Ribas L Ngo J Ortega RM García A Pérez-Rodrigo C et al. Food, youth and the Mediterranean diet in Spain: development of KIDMED. *Public Health Nutrition.* (2004) 7:931–5. 10.1079/PHN2004556 15482620

[36] Altavilla C Caballero-Pérez P. An update of the KIDMED questionnaire. *Pub Health Nutr.* (2019) 22:2543–7. 10.1017/S1368980019001058 31146796 PMC10260407

[37] Musse JP Musse N. *GENI (Gestion d’enquête nutritionnelle informatisée), version 6.* France: Micro6 (2000).

[38] Martin A. The “apports nutritionnels conseillés (ANC)” for the French population. *Reprod Nutr Develop.* (2001) 41:119–28. 10.1051/rnd:2001100 11434516

[39] EFSA. Dietary reference values for nutrients: summary report. *EFSA Supporting Pub.* (2017) 14:15121. 10.2903/sp.efsa.2017.e15121

[40] Craig CL Marshall AL Sjöström M Bauman AE Booth ML Ainsworth BE et al. International physical activity questionnaire: 12-country reliability and validity. *Med Sci Sports Exerc.* (2003) 35:1381–95. 10.1249/01.MSS.0000078924.61453.FB 12900694

[41] Van Doorn RK Winkler LM Zwinderman KH Mearin ML Koopman HM. CDDUX questionnaire for children with celiac disease. *J Pediatric Gastroenterol Nutr.* (2008) 47:147–52. 10.1097/MPG.0b013e31815ef87d 18664865

[42] Guennouni M Admou B Bourrhouate A El Khoudri N Fguirouche A Nibaruta JC et al. Quality of life of Moroccan children with celiac disease. *J Pediatric Nursing.* (2022) 62:1–7. 10.1016/j.pedn.2021.06.011 35125172

[43] Ravens-Sieberer U Gosch A Abel T Auquier P Bellach BM Bruil J et al. Quality of life in children and adolescents. *Soz Praventivmed.* (2001) 46:297–302. 10.1007/BF01321080 11759336

[44] KIDSCREEN Group. *KIDSCREEN Questionnaires: Manual and Scoring.* (n.d.). Available online at: https://www.kidscreen.org/english/analysis/evaluation-by-hand/ (Accessed July 20, 2024)

[45] Pérez-Junkera G Simón E Calvo AE Casales ZG Goicolea PO Serrano-Vela JI. Importance of an ongoing nutritional counselling intervention on eating habits of newly diagnosed children with celiac disease. *Nutrients.* (2024) 16:2418. 10.3390/nu16152418 39125299 PMC11314293

[46] Neelu, Bharti LK Samanta A Srivastava A Sarma MS Kishun J et al. Determinants of adherence to gluten-free diet in children with celiac disease and the impact of counseling by trained dietician on adherence. *JPGN Rep.* (2025) 6:473–9. 10.1002/jpr3.70073 41245033 PMC12611580

[47] Elsahoryi NA Altamimi E Subih HS Hammad FJ Woodside JV. Educational intervention improved parental knowledge, attitudes, and practices (KAP) and adherence of patients with celiac disease to a gluten-free diet. *Int J Food Sci.* (2020) 2020:8850594. 10.1155/2020/8850594 33015151 PMC7525320

[48] Muhammad N Ahad A Rashid N Gul R Tariq MA Nazir A. Assessing the impact of early nutritional intervention on pediatric celiac disease management: a prospective cohort study. *Cureus.* (2024) 16:e76059. 10.7759/cureus.76059 39835063 PMC11743799

[49] Milman M Patel M Modi A Alessi K Chauhan N Parmar MS. Celiac disease: beyond gluten—economic burdens and psychosocial impacts. *SN Compr Clin Med.* (2026) 8:11. 10.1007/s42399-025-02209-3

[50] Königová M Vňuková M Řehořková P Anders M Ptáček R. The effectiveness of gluten-free dietary interventions: a systematic review. *Front Psychol.* (2023) 14:1107022. 10.3389/fpsyg.2023.1107022 37034934 PMC10075251

[51] Razavi AC Sapin A Monlezun DJ McCormack IG Latoff A Pedroza K et al. Effect of culinary education curriculum on Mediterranean diet adherence and food cost savings in families: a randomized controlled trial. *Public Health Nutr.* (2021) 24:2297–303. 10.1017/S1368980020002256 32744215 PMC10195617

[52] Sullivan A Parsons K Therrien-Genest M Yerxa K. Cooking up knowledge: empowering high school students through a food literacy boot camp. *Am J Lifestyle Med.* (2025) 20:244–55. 10.1177/15598276251319254 39958204 PMC11826820

[53] Maddison-Roberts H Jones C Satherley R. Gluten-free diet management and well-being in children with celiac disease: a qualitative study. *Pediatr Allergy Immunol.* (2025) 36:e70061. 10.1111/pai.70061 40099805 PMC11916637

[54] Ayyıldız D Demirtaş Z Kınacı E. Psychiatric difficulties in children with celiac disease and the relationship between adherence to treatment and parental attitudes. *Turk J Gastroenterol.* (2024) 35:743–9. 10.5152/tjg.2024.23493 39344879 PMC11391222

[55] Kushkestani M Moghadassi M Sidossis L. Mediterranean lifestyle: more than a diet, a way of living (and thriving). *Endocr Metab Immune Disord Drug Targets.* (2024) 24:1785–93. 10.2174/0118715303279769240215105510 38424420

[56] N’joumi C Hamami A Elouali A Babakhouya A Rkain M. Impact of celiac disease on the quality of life of children: analysis from a Moroccan cohort. *Cureus.* (2025) 17:e89539. 10.7759/cureus.89539 40926916 PMC12415305

[57] Cheung T Setty M Fenton C McDonald CM Tsai P Lai JC et al. Neighborhood socioeconomic deprivation associates with decreased serologic normalization rates in pediatric celiac disease. *J Pediatr*. (2025) 286:114717. 10.1016/j.jpeds.2025.114717 40651553 PMC13383882

[58] Ribeiro C Pratesi C Zandonadi RP. Celiac disease and gluten-free diets: a path or barrier to food (in)security? *Nutrients.* (2025) 17:1956. 10.3390/nu17121956 40573067 PMC12195763

[59] Runde J Mears M Guandalini S Jericho H. A narrow window: booming gluten-free market and fostering healthy dietary habits in children with celiac disease. *J Pediatr Gastroenterol Nutr.* (2020) 71:533–5. 10.1097/MPG.0000000000002831 32960543

[60] Matschull L Martin NB Goday PS Chugh A. Evaluation of in-person gluten-free diet education. *JPGN Rep.* (2022) 3:e218. 10.1097/PG9.0000000000000218 37168641 PMC10158309

[61] Wieser H Ruiz-Carnicer Á Segura V Comino I Sousa C. Challenges of monitoring gluten-free diet adherence. *Nutrients.* (2021) 13:2274. 10.3390/nu13072274 34209138 PMC8308436

[62] Capra M Sguerso T Aliverti V Pisseri G Bellani A Berzieri M et al. Gluten-related nutritional challenges. *Front Nutr.* (2025) 12:1709121. 10.3389/fnut.2025.1709121 41377562 PMC12687732

[63] Hoteit M Chamas Z Assaf S Bouhairie M Bahr A Daccache R et al. Nutritional status in celiac patients in Lebanon. *F1000Research.* (2022) 11:725. 10.12688/f1000research.121859.3 37090031 PMC10119616

[64] Drabińska N Krupa-Kozak U Abramowicz P. Vitamin D and E status in children with celiac disease. *Nutrients.* (2018) 10:1768. 10.3390/nu10111768 30445682 PMC6266806

[65] Sun YH Zhou QX Tian DD Zhou JM Dong SL. Relationship between vitamin D levels and pediatric celiac disease: a systematic review and meta-analysis. *BMC Pediatr.* (2024) 24:185. 10.1186/s12887-024-04688-0 38491474 PMC10943820

[66] Zablotsky B Ng A Black L Haile G Bose J Jones J et al. Associations between screen time use and health outcomes among US teenagers. *Prev Chronic Dis.* (2025) 22:E38. 10.5888/pcd22.240537 40638804 PMC12249308

[67] Nestares T Martín-Masot R De Teresa C Bonillo R Maldonado J Flor-Alemany M et al. Mediterranean diet and bone health in celiac children. *Nutrients.* (2021) 13:1636. 10.3390/nu13051636 34068001 PMC8152289

[68] Cascobelo-Águeda L Garrido-Bueno M Rodríguez-García M Tirado-Hernández P Andrade-Gómez E Fagundo-Rivera J et al. The multidimensional impact of gluten-free diet adherence on quality of life in pediatric and adolescent celiac disease: a systematic review. *Children (Basel).* (2026) 13:722. 10.3390/children13060722 42353892 PMC13297346

[69] Chellan D Muktesh G Vaiphei K Berry N Dhaka N Sinha SK et al. Effect of gluten-free diet and compliance on quality of life in pediatric celiac disease patients. *JGH Open.* (2019) 3:388–93. 10.1002/jgh3.12172 31633043 PMC6788369

[70] Gunnoo S Siddiqui S Toorabally Z Mahomoodally MF Alrefaei AF Jeewon R. Mediterranean diet: potential health benefits, barriers to adherence and intervention strategies. *Curr Res Nutr Food Sci.* (2026) 14: 53–71. 10.12944/CRNFSJ.14.1.4

[71] Jennings A Shannon O Gillings R Lee V Elsworthy R Bundy R et al. Effectiveness and feasibility of a theory-informed intervention to improve Mediterranean diet adherence, physical activity and cognition in older adults at risk of dementia: the MedEx-UK randomized controlled trial. *BMC Med.* (2024) 22:600. 10.1186/s12916-024-03815-z 39716203 PMC11667912

[72] Silva P. Enhancing adolescent food literacy through Mediterranean diet principles: from evidence to practice. *Nutrients.* (2025) 17:1371. 10.3390/nu17081371 40284234 PMC12030646

[73] Kontele I Panagiotakos DB Yannakoulia M Vassilakou T. Socio-demographic determinants of Mediterranean diet adherence: results of the EU-National Health Interview Survey (EHIS-3). *J Hum Nutr Diet.* (2025) 38:e70023. 10.1111/jhn.70023 39934642 PMC11814368

[74] Lenzi F De Falco C Cavagnuolo M Esposito V Iazzetta F. Toward an integrated model of dietary behavior: social determinants and adherence to the Mediterranean diet. *PLoS One.* (2025) 20:e0328853. 10.1371/journal.pone.0328853 41406090 PMC12711094

[75] Gharbi A Arfaoui M Boudiche S Ameur M. Dietary patterns and socioeconomic factors affecting Mediterranean diet adherence in Tunisia. *New Medit.* (2025) 24:89–107. 10.30682/nm2502f

[76] Baltaci A Laska MN Horning M Hearst M Lee J Fulkerson JA. Parent meal self-efficacy and practices in households with healthy home food environments in the face of economic hardship. *Appetite.* (2023) 190:107029. 10.1016/j.appet.2023.107029 37683896 PMC10543555

[77] Missbach B Schwingshackl L Billmann A Mystek A Hickelsberger M Bauer G et al. Gluten-free food database: the nutritional quality and cost of packaged gluten-free foods. *PeerJ*. (2015) 3:1337. 10.7717/peerj.1337 26528408 PMC4627916

[78] Mármol-Soler C Matias S Miranda J Larretxi I Fernández-Gil MP Bustamante M et al. Gluten-free products: do we need to update our knowledge? *Foods*. (2022) 11:3839. 10.3390/foods11233839 36496647 PMC9735448

