# Supplementary material for: Impact of Mediterranean diet–based nutritional intervention on gluten-free diet, nutritional status, and quality of life in a pediatric celiac population: a randomized controlled trial
Source: Front Nutr. 2026 Aug 28;13:1878608. doi: 10.3389/fnut.2026.1878608 (PMC13561891; doi:10.3389/fnut.2026.1878608)
Supplement: Supplementary file 1 [file Table_1.DOC]

Demographics and anthropometrics characteristics of non-participants celiac pediatric patients

| N | 54 |
| --- | --- |
| Female n (%) | 27(50) |
| **Mean age (years)** | 10±4 |
| **Age at onset GFD (years)** | 5±4 |
| **Duration of GFD (years)** | 4±3 |
| **Household food budget n (%)** |  |
| ≤ 21500 DZD | 18 (33) |
| > 21500 DZD | 36 (67) |
| **Gluten-free food budget n (%)** |  |
| ≤ 5000 DZD | 23 (43) |
| > 5000 DZD | 31 (57) |
| **Anthropometric parameters** |  |
| **Weight (kg)** | 27.49±11.97 |
| **Height (m)** | 1.26±0.28 |
| **BMI (kg/m^2^)** | 15.91±2.27 |
| Underweight n (%) | 13 (24) |
| Normal weight n (%) | 38 (70) |
| Overweight/Obesity n (%) | 3 (6) |

n: Number of patients; DZD: National currency; GFD: Gluten-free diet. Results are presented as mean ± standard deviation and percentages for a total of 54 children and adolescents with celiac disease
